# Uncovering the influence of social media marketing activities on Generation Z’s purchase intentions and eWOM for organic cosmetics

**DOI:** 10.1371/journal.pone.0325953

**Published:** 2025-06-11

**Authors:** Gia Khuong An, Thi Thuy An Ngo

**Affiliations:** 1 Department of International Business, FPT University, Can Tho City, Vietnam; 2 Department of Business Administration, FPT University, Can Tho City, Vietnam; SEGi University: SEGi University Kota Damansara, MALAYSIA

## Abstract

The organic cosmetics market in Vietnam is rapidly growing, especially among Generation Z consumers who prioritize sustainability and eco-friendly products. Despite this expansion, the key factors driving purchasing decisions for organic cosmetics have not been adequately researched. This study addresses this gap by examining the influence of Social Media Marketing Activities (SMMAs) on Generation Z’s purchase intentions and eWOM, with perceived quality and perceived value as mediating factors. Using a quantitative approach, data were collected from 315 Generation Z participants in Vietnam through a structured questionnaire. The study explores various dimensions of SMMAs—interaction, customization, trendiness, and entertainment—and their effects on perceived quality, perceived value, eWOM, and purchase intention. Results show that SMMAs have a stronger impact on perceived quality (β = 0.726) than on perceived value (β = 0.503), suggesting that social media marketing strategies are particularly effective in shaping how Generation Z evaluates product quality. Additionally, perceived quality significantly influences perceived value (β = 0.312), eWOM (β = 0.346), and purchase intention (β = 0.279). Similarly, perceived value positively impacts eWOM (β = 0.395) and purchase intention (β = 0.402), while eWOM itself plays a direct role in driving purchase intention (β = 0.167). These findings highlight the interconnected role of social media marketing in influencing consumer behavior through both direct and mediated effects. This research provides strategic direction for brands in Vietnam’s organic cosmetics sector targeting Generation Z. By leveraging these findings, brands develop targeted social media campaigns that emphasize quality perceptions through interactive content and customized experiences, thereby effectively driving both purchase decisions and positive eWOM among Generation Z consumers.

## 1. Introduction

The organic cosmetics industry has grown significantly globally, driven by increasing consumer awareness of health and environmental sustainability [1]. In Vietnam, this market segment shows promising potential, fueled by a growing middle class and increasing consumer demand for natural and eco-friendly products [2]. Among the various demographic groups contributing to this growth, Generation Z stands out due to its digital nativity and its considerable influence on market dynamics and consumption trends [3]. As this generation’s purchasing behaviors are largely shaped by their interactions on social media, understanding the role of Social Media Marketing Activities (SMMAs) in influencing their purchase decisions becomes crucial for businesses targeting this segment [4]. This global shift towards organic cosmetics aligns with broader sustainability trends, where consumers increasingly demand transparency in ingredient sourcing, ethical production practices, and environmental responsibility [5]. These trends are particularly pronounced in digital spaces, where Generation Z actively seeks and shares information about sustainable products, creating a unique intersection of sustainability consciousness and digital engagement [6].

Organic cosmetics, typically defined as beauty products derived from natural and minimally processed ingredients, have garnered attention for their perceived health benefits and environmentally conscious production processes [7]. Despite being in its nascent stages, the organic cosmetics market in Vietnam exhibits strong growth potential [8]. However, there remains a significant gap in the literature concerning the factors that drive Generation Z’s purchase intentions and their propensity to engage in electronic word-of-mouth (eWOM) regarding organic cosmetics, particularly in the Vietnamese context. This gap may stem from various barriers in current marketing practices, such as a lack of authenticity in brand messaging [9], limited consumer trust in organic certification standards [10], or cultural preferences influencing online engagement [11]. Addressing these factors will help to contextualize the study’s significance within Vietnam’s unique digital and consumer landscape.

Social Media Marketing Activities (SMMAs) have been extensively explored across different industries, including fashion and consumer goods, due to their growing impact on consumer behavior. For instance, Zeqiri et al. [12] investigated the role of SMMAs in the fashion industry and found that they significantly boost brand equity, which in turn positively affects purchase intention. Similarly, Van and Fah [13] examined SMMAs among Vietnamese millennials, concluding that SMMAs enhance brand loyalty through the mediating effects of brand trust, brand image, and self-congruence. In the cosmetics industry, the role of SMMAs has also been highlighted. Choedon and Young-Chan [14] found that SMMAs significantly increase brand awareness and purchase intention among Generation Z consumers in the organic cosmetics sector. Additionally, Ananthsai et al. [15] emphasized the effectiveness of interactive SMMAs in promoting skincare products, showing that engaging content can enhance consumer interaction and brand affinity. However, these studies often overlooked the unique dynamics of organic cosmetics and the specific behaviors of Generation Z—a demographic that is not only digitally savvy but also highly critical of marketing, especially when it comes to authenticity and sustainability. Global research indicates that Generation Z consumers consistently demonstrate strong preferences for sustainable products while maintaining high levels of social media engagement [16]. These patterns highlight the timely nature of examining how digital marketing strategies can effectively connect with this sustainability-conscious, digitally native generation.

In addition to the role of SMMAs, factors such as perceived quality and perceived value have been identified as key mediators in shaping consumer behavior. For example, Banerji and Singh [17] found that perceived quality mediates the relationship between marketing activities and customer loyalty in e-commerce. Similarly, Alkufahy et al. [18] demonstrated that perceived value significantly mediates the impact of e-marketing on customer satisfaction and loyalty. Despite these insights, the interplay between SMMAs, perceived quality, and perceived value in the context of Generation Z’s behavior toward organic cosmetics has not been comprehensively examined, particularly within the Vietnamese market.

Furthermore, while existing literature provides valuable insights into SMMAs, perceived quality, and perceived value, there is a notable research gap concerning their combined effects on purchase intention and eWOM for organic cosmetics. This gap is particularly significant given the unique characteristics of the Vietnamese market, such as differing levels of trust in organic product claims [19], the influence of cultural norms on social media engagement [11], and the evolving nature of digital marketing strategies aimed at Generation Z as a consumer segment [20]. Therefore, the purpose of this study is to investigate the influence of Social Media Marketing Activities on Generation Z’s purchase intention and electronic word-of-mouth for organic cosmetics in Vietnam, with perceived quality and perceived value as mediating factors. Through focusing on this specific demographic and product category in the Vietnamese market, this research aims to provide novel insights that bridge the existing knowledge gap. The study’s findings will contribute to the body of literature on consumer behavior in emerging markets and offer practical implications for businesses seeking to effectively market organic cosmetics to Generation Z consumers in Vietnam through social media platforms.

This research is particularly relevant given the global shift towards sustainable consumption and digital marketing innovation. While worldwide trends show increasing integration of sustainability messaging in social media marketing, the unique characteristics of Vietnam’s market require a specialized understanding of how these global trends can be effectively localized. The research differentiates itself by focusing specifically on the organic cosmetics market in Vietnam, an area that has received limited attention in previous studies. Moreover, it integrates the constructs of SMMAs, perceived quality, and perceived value to provide a holistic understanding of the factors driving Generation Z’s purchase intentions and engagement with eWOM. Finally, by examining the behaviors and preferences of Generation Z in an emerging market setting, the study contributes to a more nuanced understanding of this influential consumer group, offering practical implications for businesses aiming to utilize social media marketing strategies to reach this target audience effectively.

## 2. Literature review

### 2.1. Theoretical background

#### 2.1.1. *Organic Cosmetics.*

Organic cosmetics refer to personal care products formulated with natural, organically sourced ingredients, excluding synthetic chemicals, parabens, sulfates, and other harmful additives [21]. These products are perceived as safer and more skin-friendly, making them a preferred choice for individuals prioritizing wellness, particularly Generation Z [22]. Moreover, a recent study by Limbu and Ahamed [23] found that consumers’ purchase intentions for organic cosmetics are significantly influenced by health consciousness, environmental concern, and product quality. Similarly, Vergura et al. [24] found that consumer knowledge about organic personal care products and their sensory appeal positively impacts attitudes, thereby enhancing purchase intentions. Additionally, Ruslim et al. [25] identified key determinants—such as attitudes, subjective norms, and perceived behavioral control—as significant predictors of purchase intention for organic skincare products. These factors resonate closely with the values of Generation Z, further solidifying organic cosmetics as a preferred choice among this demographic.

Recent studies have further highlighted the importance of natural antioxidants in organic cosmetics. These antioxidants, derived from plant extracts, provide significant protective benefits against environmental stress and aging, enhancing product appeal to health-conscious consumers [26]. Additionally, advancements in biotechnology have supported the extraction and formulation of bio-based ingredients, a trend driven by consumer demand for sustainable, eco-friendly alternatives [27]. This shift in consumer preferences is particularly relevant in Vietnam, where the organic cosmetics market is experiencing rapid growth driven by heightened environmental awareness and government regulations promoting sustainable and eco-friendly practices [28]. As the global trend toward “green beauty” continues, the Vietnamese market shows substantial growth potential, driven by consumers’ increasing demand for products with minimal environmental impact [29]. This growth mirrors international movements towards sustainability and offers a unique opportunity to understand how organic cosmetics resonate with Generation Z in Vietnam. Despite the extensive research on organic cosmetics, there is a limited exploration of interdisciplinary perspectives, such as behavioral economics and cultural studies, which could provide deeper insights into consumer motivations and purchase decisions. For instance, behavioral economics can help explain how cognitive biases and heuristics influence organic cosmetics purchases [30], while cultural studies can shed light on how social and cultural values shape consumer preferences across different regions [31].

#### 2.1.2. *Social Media Marketing Activities (SMMAs).*

Social Media Marketing Activities (SMMAs) refer to the strategic use of social media platforms to promote products, services, or brands. SMMAs have become essential components of modern marketing, especially when targeting Generation Z, a demographic known for its active presence on social media and digital nativity [32]. Research consistently highlights that effective SMMAs enhance consumers’ perceptions of brand quality and value, leading to stronger brand relationships, loyalty, and increased purchase intentions [33,34]. Additionally, by showcasing customer reviews and influencer endorsements, SMMAs significantly influence Generation Z’s purchasing decisions [17], further reinforcing the critical role social media plays in modern consumer behavior.

Previous studies have classified the components of SMMAs into several dimensions. In research on luxury brands, Godey et al. [35] and Kim and Ko [36] identified five key components of SMMAs: entertainment, interaction, trendiness, customization, and word of mouth (WOM). This classification has been applied across various sectors. For example, Jamil et al. [4] used these components to explore SMMAs in online marketplaces, while Seo and Park [37] expanded the framework by adding perceived risk in a study on the airline industry. Similarly, Sano [38] applied four components—interaction, trendiness, customization, and perceived risk—to the insurance sector, further broadening the applicability of SMMAs to different industries. In the context of organic cosmetics, this study adopts four key components of SMMAs—entertainment, interaction, trendiness, and customization—similar to the approaches used in recent studies by Anas et al. [39] in the restaurant industry and Grari and Kaplan [40] in the apparel sector. These components reflect the diverse strategies brands use to engage consumers on social media platforms.

For organic cosmetics brands, social media is a critical tool for reaching younger audiences. Generation Z, in particular, is highly influenced by digital content, peer reviews, and influencer endorsements [41]. With rising consumer demand for natural and ethical products, organic cosmetics brands are increasingly utilizing social media to communicate their product quality and environmental values. SMMAs enable brands to engage with eco-conscious consumers, offering interactive content and fostering direct brand-consumer engagement [42]. This engagement has been shown to positively influence purchase intentions and generate electronic word-of-mouth [43]. However, prior studies on SMMAs have predominantly focused on their direct effects on consumer engagement and purchase intention, with limited attention to potential moderating and mediating factors such as cultural differences and digital literacy levels [44]. Future research could benefit from integrating perspectives from cultural studies and behavioral economics to explore how cognitive and social influences shape the impact of SMMAs on Generation Z consumers [4].

In summary, SMMAs play a vital role in shaping consumer perceptions and driving purchasing behavior, especially among Generation Z. By leveraging these digital marketing activities, organic cosmetics brands can build stronger relationships with their target audiences, ultimately enhancing their market presence in an increasingly competitive industry.

### 2.2. Hypothesis development

#### 2.2.1. SMMAs and perceived quality.

Social Media Marketing Activities (SMMAs) are an essential component of modern marketing, particularly for engaging younger, digitally savvy consumers. Recent empirical research demonstrates the significant impact of SMMAs on consumers’ perceptions of product quality. For instance, Cheung et al. [45] found that interactive and engaging social media content can enhance perceived quality, as seen in their study of smartphone brands. This interactive engagement not only elevates consumers’ quality perceptions but also strengthens consumer-brand relationships. Similarly, Hafez [46] explored the influence of SMMAs on brand equity in the banking sector and found that SMMAs significantly enhance perceived quality through the mediating role of brand experience. Furthermore, Jamil et al. [4] examined the relationship between SMMAs, consumer intentions, and satisfaction, revealing that effective SMMAs positively influence both perceived quality and customer satisfaction.

In the cosmetics industry, Man and Rahman [47] showed that effective SMMAs significantly influence perceived quality, particularly among college students, leading to greater brand loyalty. Their research indicates that emphasizing product benefits and quality on social media leads to increased consumer trust and more favorable brand perceptions. Additionally, Sharma and Foropon [48] emphasized the importance of social media content focusing on natural ingredients and eco-friendly practices, particularly in the organic cosmetics sector. Their findings indicate that social media posts emphasizing these aspects can enhance the perceived quality of organic products, especially among health-conscious and environmentally aware Generation Z consumers. Despite these findings, the degree to which SMMAs influence perceived quality may vary depending on cultural and regional factors. For example, in Vietnam, Generation Z consumers are highly active on social media and often rely on user-generated content and influencer recommendations when assessing product quality [49]. The prevalence of counterfeit cosmetics in Vietnam further heightens the importance of perceived quality [50], making social media a critical platform for brands to communicate product authenticity and effectiveness.

Collectively, these studies highlight the critical role of SMMAs in shaping consumers’ perceptions of quality, especially in the organic cosmetics market. Given that Generation Z in Vietnam is increasingly driven by health and environmental concerns, it is reasonable to expect that SMMAs will significantly influence their perception of organic cosmetics quality. Therefore, the following hypothesis is proposed:

**H1:** SMMAs have a significant positive influence on perceived quality.

#### 2.2.2. *SMMAs and Perceived Value.*

Social Media Marketing Activities (SMMAs) play a pivotal role in shaping perceived value, which is defined as the consumer’s assessment of a product’s worth based on anticipated benefits. Research by Rahman et al. [51] and Yadav and Rahman [52] provides strong evidence that social media marketing significantly enhances perceived value, particularly for eco-friendly products. Their studies underscore the importance of using social media to communicate the long-term sustainability and ethical benefits of products, which in turn elevate perceived value.

In the cosmetics industry, Bushara et al. [43] and Choedon and Lee [14] demonstrated that SMMAs focusing on product benefits and social responsibility positively influence perceived value, particularly among younger consumers. By highlighting ethical production practices and tangible benefits, such as skin health or reduced exposure to chemicals, brands can foster a stronger sense of value in the eyes of consumers. This is further supported by Pop et al. [53], who found that social media campaigns promoting the long-term benefits of organic cosmetics—such as better skin health and environmental protection—significantly increased perceived value among consumers. However, perceived value is influenced by more than just product attributes, it also depends on the credibility of marketing messages and the extent to which consumers trust brand communications [54]. In Vietnam, where skepticism toward exaggerated marketing claims is prevalent, brands must ensure that SMMAs provide transparent, educational content rather than solely promotional messaging [55]. Authentic storytelling, customer testimonials, and interactive engagement may enhance perceived value by reinforcing trust and demonstrating the tangible benefits of organic cosmetics [56].

For Generation Z consumers in Vietnam, who are deeply concerned about both their health and environmental sustainability, the perceived value of organic cosmetics is closely linked to the information they receive through SMMAs. Therefore, using social media to emphasize the health and environmental benefits of organic cosmetics is likely to enhance perceived value among this demographic. Hence, the following hypothesis is proposed:

**H2:** SMMAs have a significant positive influence on perceived value.

#### 2.2.3. *Perceived Quality and Perceived Value.*

Perceived quality refers to a consumer’s evaluation of a product’s overall excellence or superiority. [57]. It plays a critical role in shaping consumers’ perceived value, particularly in product categories where consumers are more discerning about attributes such as sustainability and effectiveness. When customers believe a product to be of high quality, they are more inclined to link it with higher value, as the quality attributes serve as a foundation for evaluating the product’s worth [58]. Osburg et al. [59] highlighted that in emerging markets, consumers tend to place greater emphasis on perceived quality when assessing product value, especially for sustainable luxury products. This is particularly relevant in markets like Vietnam, where Generation Z consumers are increasingly drawn to eco-conscious products such as organic cosmetics.

Moreover, Hudayah et al. [60] found that perceived quality is a significant predictor of perceived value among Generation Z, especially for green products. Their research indicates that this generation tends to see quality as a key factor in determining the value of products with ethical or sustainable attributes. This underscores the critical role of perceived quality in influencing the perceived value of green products among young consumers. In the organic cosmetics industry, Ahmad and Omar [61] showed that when Generation Z consumers perceive organic cosmetics as high quality, they are more inclined to view these products as valuable. Their study highlights the importance of maintaining high quality standards to enhance the perceived value and the purchase intention of this consumer segment. This relationship emphasizes the critical role of quality in influencing perceived value, particularly for marketers aiming to attract and retain Generation Z consumers in the organic cosmetics sector. Therefore, the following hypothesis is proposed:

**H3:** Perceived quality has a significant positive influence on perceived value.

#### 2.2.4. Perceived Quality and Electronic Word-of-Mouth.

Perceived quality not only enhances perceived value but also plays a pivotal role in driving electronic word-of-mouth (eWOM). When consumers recognize a product as high quality, they are more inclined to share positive experiences and recommendations online. eWOM, particularly among Generation Z, is highly influenced by perceptions of quality, as this demographic tends to engage in online discussions and reviews when they feel confident in the product’s excellence [62]. Research by Ngo et al. [63] found that information quality, a component of perceived quality, has the strongest positive influence on both information adoption and information usefulness, which in turn drive eWOM and purchase intentions. Similarly, Tabassum et al. [64] highlighted that high-quality narrative advertisements and eWOM significantly impact Generation Z’s purchase intentions, emphasizing the role of perceived quality in these processes. In Vietnam, where the organic cosmetics market is still developing, Generation Z consumers rely heavily on peer reviews and influencer endorsements to validate product quality [65]. Given the widespread issue of counterfeit products, eWOM serves as a critical mechanism for consumers to distinguish between trustworthy brands and low-quality alternatives [66].

Xiao et al. [67] further demonstrated that high-quality of product information on social platforms enhances eWOM, with consumers more likely to engage in sharing product experiences when they perceive the information as credible and valuable. Another study by Tong and Su [68] revealed that perceived quality positively influences consumer trust, further supporting the vital function of perceived quality in driving eWOM behaviors. In the case of organic cosmetics, Nguyen et al. [69] provided evidence that perceived quality of green cosmetics directly affects consumers’ attitude and their likelihood of engaging in positive eWOM. These findings collectively highlight the importance of perceived quality in fostering positive eWOM for organic cosmetics among Generation Z consumers. Therefore, the following hypothesis is proposed:

**H4:** Perceived quality has a significant positive influence on electronic word-of-mouth.

#### 2.2.5. Perceived quality and purchase intention.

Perceived quality is a significant determinant of purchase intention, particularly in markets where consumers prioritize sustainability and product efficacy, such as the organic cosmetics industry [70]. For Generation Z, who are highly conscious of environmental and ethical considerations, perceived quality plays an even more prominent role in their purchasing decisions [71]. When they believe that a product meets high standards of quality—whether in terms of ingredients, production processes, or overall effectiveness—they are more likely to develop strong purchase intentions.

Dragolea et al. [72] found that Generation Z consumers are more likely to purchase organic cosmetics when they perceive these products to be of superior quality. Key factors such as ingredient purity, product safety, and effectiveness drive this demographic’s purchase behavior, particularly in green product sectors [23,73] Specifically, Limbu and Ahamed [23] highlights that perceived quality is a critical determinant of purchase intention in the green cosmetics sector. Their systematic review reveals that high-quality attributes significantly enhance the likelihood of purchase. Similarly, Zhuang et al. [73] found that perceived quality, along with other cognitive factors, significantly boosts green purchase intentions. Their meta-analysis approach provides robust evidence that perceived quality is a pivotal factor in the decision-making process of Generation Z consumers. In the Vietnamese market, trust in organic cosmetics is closely linked to the perceived authenticity of quality claims [74]. Brands that provide detailed, science-backed explanations of ingredient efficacy and conduct third-party product testing are more likely to convert quality perceptions into purchase intentions [75]. These findings underscore the pivotal role of perceived quality in shaping the purchasing decisions of Generation Z, suggesting that marketers should emphasize quality attributes in their promotional strategies to effectively target this consumer segment. Therefore, the following hypothesis is proposed:

**H5:** Perceived quality has a significant positive influence on purchase intention.

#### 2.2.6. Perceived value and electronic Word-of-Mouth.

Perceived value is a critical factor influencing electronic word-of-mouth (eWOM) among Generation Z consumers, particularly in the organic cosmetics market. Recent studies have highlighted that perceived value, which encompasses the benefits and overall worth that consumers associate with a product, significantly impacts their willingness to share positive experiences online. Specifically, Lin et al. [76] found that perceived value directly enhances consumers’ intention to engage in eWOM by increasing their satisfaction with the product. Similarly, Limbu and Ahamed [23] demonstrated that the perceived value of green cosmetics positively correlates with consumers’ eWOM behaviors, driven by their appreciation for the product’s environmental benefits and quality.

Moreover, the research by Nguyen et al. [77] highlighted that perceived value acts as a mediator in the relationship between consumer trust and eWOM, suggesting that higher perceived value leads to greater consumer confidence in the product, leading to more frequent eWOM participation. This finding is supported by the work of Panopoulos et al. [78], who showed that the perceived value of green products significantly influences Generation Z’s online reviews and recommendations, as these consumers are particularly attuned to the ethical and health-related benefits of such products. Additionally, the study by Yu et al. [79] highlighted that perceived value enhances the emotional connection between consumers and organic brands and effectively motivates customer engagement behavior, including eWOM. For Vietnamese Generation Z consumers, perceived value is shaped not only by product efficacy but also by brand authenticity and alignment with social values [80]. This generation is highly receptive to cause-driven marketing, meaning that brands promoting sustainability and ethical production can amplify eWOM by fostering a sense of social responsibility among consumers. Therefore, the following hypothesis is proposed:

**H6:** Perceived value has a significant positive influence on electronic word-of-mouth.

#### 2.2.7. *Perceived value and purchase intention.*

Perceived value is a crucial factor influencing the purchase intentions of Generation Z consumers [59], particularly in the context of organic cosmetics. Defined as the consumer’s evaluation of the benefits versus the costs of a product, perceived value plays a pivotal role in decision-making processes [81]. Zhuang et al. [73] demonstrated that green perceived value has a significant positive impact on green purchase intention among Generation Z. This study indicates that when Generation Z consumers perceive high value in environmentally friendly products, their intention to buy these products increases. Similarly, Hudayah et al. [60] identified environmental concern, conditional value and functional value as key drivers of Generation Z’s purchase decisions regarding green products.

Furthermore, a study by Saut and Saing [82] supports the notion that environmentally friendly products, perceived as offering high value, are more inclined to be purchased by environmentally conscious consumers. This study emphasizes that Generation Z consumers prioritize products that align with their environmental values and offer tangible benefits. For organic cosmetics specifically, Limbu and Ahamed [23] emphasized that Generation Z’s purchase intentions for organic cosmetics are significantly driven by their perceived value, including functional and environmental benefits. This systematic review underscores the importance of perceived value in shaping the purchase intentions of Generation Z consumers towards organic cosmetics, highlighting that both functional benefits and environmental benefits play crucial roles in their decision-making process. For organic cosmetics brands targeting Vietnamese Generation Z, perceived value is enhanced when marketing efforts emphasize cost-effectiveness in addition to sustainability benefits. While this cohort values environmental consciousness, they are also price-sensitive and look for tangible justifications for premium pricing [83]. Offering educational content on product longevity, multi-purpose usage, and long-term skin benefits can strengthen perceived value and drive purchase intention [74]. These findings highlight the role of perceived value in driving Generation Z consumers’ purchasing intentions for organic cosmetics. Therefore, the following hypothesis is proposed:

**H7:** Perceived value has a significant positive influence on purchase intention.

#### 2.2.8. *Electronic Word-of-Mouth and purchase intention.*

Electronic word-of-mouth (eWOM) is another critical factor that significantly impacts the purchase intentions of Generation Z consumers. In the digital age, eWOM—comprised of online reviews, recommendations, and social media interactions—shapes consumer perceptions and plays a key role in influencing purchase decisions [84]. Research by Xiao et al. [67] indicates that the structure and credibility of eWOM significantly affect Generation Z’s purchase intentions by improving their perceptions of product quality and trust. This demographic is highly reliant on digital information, and the credibility of online reviews holds substantial weight in their decision-making processes. Similarly, studies by Ismagilova et al. [85] revealed that argument quality and the valence (positive or negative tone) of eWOM are strong predictors of purchase intention, especially among younger consumers who often consult online feedback before making a purchase.

Moreover, Boateng [86] emphasizes that eWOM credibility and emotional trust play crucial roles in purchase decisions, highlighting the importance of trustworthy online information. Beyari and Garamoun [87] also confirm that eWOM positively influences purchase intention by increasing perceived usefulness and reducing perceived risk. Positive reviews not only improve the perceived quality of the product but also mitigate concerns related to product performance or authenticity, thereby encouraging consumers to proceed with their purchase [88]. Additionally, Sylvia and Ramli [89] highlighted that positive eWOM enhances both brand image and purchase intention, particularly in the skincare industry, where trust and reputation are crucial. Given the high engagement of Vietnamese Generation Z consumers with social media, eWOM is a dominant factor in their purchase decision-making process [90]. Influencer endorsements, in particular, carry substantial weight in validating product credibility [91]. Collectively, these findings emphasize the critical role of eWOM in driving purchase intentions for organic cosmetics among Generation Z consumers. Therefore, the following hypothesis is proposed:

**H8:** Electronic word-of-mouth has a significant positive influence on purchase intention.

### 2.3. Research framework

**Fig 1:**
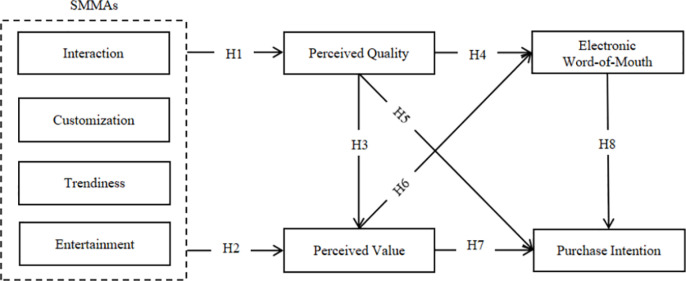
The proposed research framework.

The theoretical framework (Figure 1) illustrates the hypothesized relationships between Social Media Marketing Activities (SMMAs) and their impact on purchase intention and electronic word-of-mouth (eWOM) for organic cosmetics among Generation Z consumers in Vietnam. The model proposes that SMMAs, comprising interaction, customization, trendiness, and entertainment, influence perceived quality and perceived value. These perceptions act as mediators in the framework. Perceived quality is hypothesized to affect perceived value, electronic word-of-mouth, and purchase intention. Similarly, perceived value is proposed to influence both electronic word-of-mouth and purchase intention. Lastly, the model suggests a relationship between electronic word-of-mouth and purchase intention. This framework aims to elucidate the complex interplay between social media marketing efforts and consumer behavior in the context of organic cosmetics, with a specific focus on the Generation Z demographic in Vietnam.

## 3. Methodology

To explore the influence of Social Media Marketing Activities (SMMAs) on Generation Z’s purchase intention and electronic word-of-mouth (eWOM) for organic cosmetics in Vietnam, with perceived quality and value as mediators, this study employed a quantitative research design. The analysis was conducted using Partial Least Squares Structural Equation Modeling (PLS-SEM) via SmartPLS software, which was chosen for its capacity to handle complicated models with multiple constructs as well as its ability to manage non-normal data distributions, a common issue in social science research.

### 3.1. Participants

The study focused on Vietnamese Generation Z consumers who are actively engaged with social media and have expressed interest in organic cosmetics. This demographic is increasingly influential in the organic cosmetics market, as noted by Tsai and Tang [92]. A total of 315 valid responses were collected via an online survey, distributed through various social media platforms to maximize reach and participant diversity. The respondents reflected a broad cross-section of Generation Z, encompassing various genders, ages and income levels. This diversity was essential to enhance the generalizability of the findings to the wider Generation Z population in Vietnam. The demographic information of the participants is detailed in Table 1.

**Table 1:** Demographic profile of respondents.

Demographic Information (N = 315)		Frequency	Percentage
Gender	Male	142	45.08%
	Female	173	54.92%
Age	18 to 20 years old	128	40.63%
	21 to 24 years old	170	53.97%
	25 to 29 years old	17	5.40%
Monthly income	Less than 5 million VND	248	78.73%
	From 5 to 10 million VND	43	13.65%
	From 10 to 20 million VND	14	4.44%
	From 20 to 30 million VND	8	2.54%
	More than 30 million VND	2	0.64%

### 3.2. Instruments

The primary instrument for data collection was a structured questionnaire, developed through a rigorous process involving an extensive literature review and expert consultations to ensure validity and reliability. The questionnaire was designed in distinct sections to gather detailed and relevant information for the study. Further details regarding the questionnaire are provided in the supporting information (see S1 File).

The first section served as a screening segment, providing a definition of organic cosmetics and including a question to confirm whether participants had ever purchased or used organic cosmetics, ensuring their familiarity with the subject. The second section collected demographic data such as gender, age, and monthly income, which were essential for understanding participant backgrounds and offering critical context for analyzing behavioral differences among subgroups within Generation Z. The subsequent sections concentrated on measuring the core constructs of the research model. The assessment of Social Media Marketing Activities (SMMA) was conducted using a multi-dimensional scale with items drawn from established literature. Interaction, customization, and trend were each assessed using 3-item scales adapted from Malarvizhi et al. [93] and Yadav and Rahman [52], while entertainment was evaluated using a 3-item scale based on Bushara et al. [43] and Zarei et al. [94]. Perceived quality was measured using a 3-item scale adapted from Suhud et al. [95] and Zeithaml [57]. Perceived value was assessed using a 3-item scale from Bushara et al. [43] and Doszhanov and Ahmad [96]. Electronic Word of Mouth (eWOM) was examined using a 3-item scale adapted from Mim et al. [97]. Finally, purchase intention was assessed using a 4-item scale adapted from Alalwan [98]. These measurement scales were selected due to their demonstrated reliability and validity in previous studies, ensuring their suitability for the current study’s context. Utilizing these established scales ensured consistency in capturing the key variables in this research on social media marketing and consumer behavior.

### 3.3. Data collection

The data for this study was collected through a quantitative research approach using an online survey administered via Google Forms. The choice of Google Forms was based on its accessibility and ease of use, as highlighted by Vasantha Raju and Harinarayana [99], which allowed for efficient and effective data collection. The study adhered to ethical standards by obtaining approval from the Board of Directors of FPT Can Tho University (Approval No. 20240603.07) and following best practices for online survey research [100]. Informed consent was collected from all participants, ensuring they were fully informed about the study’s purpose, their rights, and the use of their information strictly for research purposes. Participants were assured of their voluntary, privacy and anonymity, maintaining the ethical integrity of the research throughout the process.

The sample size determination followed Hair et al.’s [101] guideline, which recommends a minimum of 250 participants for models with 25 observed variables, applying a 10:1 ratio. Data collection took place from September 10 to September 26, 2024, resulting in a final sample of 315 respondents. This exceeded the recommended threshold, ensuring sufficient statistical power for the analysis. The sample size of 315 respondents was determined based on statistical recommendations for PLS-SEM analysis, as prior research suggests that a minimum sample size of 300 is often recommended to ensure statistical power and mitigate risks such as false positives or underpowered results [102]. The questionnaire used for data collection is provided in the supporting information (see S1 File), and the dataset is available in S2 File. Besides, the demographic distribution of respondents was monitored during data collection to ensure diversity across age groups, income levels, and geographic locations, increasing the applicability of the findings. While this sample does not allow for full population-wide inference, it provides meaningful insights into consumer trends within the target segment.

A non-probability convenience sampling technique was employed due to its efficiency and practicality in reaching the target demographic [103]. While this method is widely used in social media marketing research, it has inherent limitations, including potential selection bias and limited representativeness [104]. Since convenience sampling does not provide equal selection probability for all individuals in the population, the findings must be interpreted with caution regarding generalizability. To mitigate these concerns, we recruited participants through multiple online platforms frequently used by Generation Z in Vietnam, ensuring a broad representation of social media users. This approach helped capture a broad range of social media users and minimized the risk of skewed responses. Additionally, clear participant selection criteria such as active engagement with social media marketing content, were implemented to enhance the relevance of the sample.

### 3.4. Data analysis

The collected data were analyzed using Partial Least Squares Structural Equation Modeling (PLS-SEM) with SmartPLS software. PLS-SEM was chosen for its capacity to handle complex models and its robustness in delivering reliable results, even with smaller sample sizes and non-normal data distributions [105]. This method is particularly suitable for prediction-oriented research like this study, as it focuses on maximizing explained variance among dependent variables rather than solely examining model structure [106]. Furthermore, PLS-SEM’s computational efficiency makes it ideal for analyzing intricate relationships within models containing multiple constructs and indicators [107]. Besides, PLS-SEM has the potential to address the challenges posed by non-probability sampling methods by providing robust statistical insights despite potential biases in sample representativeness [108]. Its flexibility in handling real-world data complexities ensures that findings are both reliable and actionable.

To verify the reliability of the constructs, both Cronbach’s Alpha (CA) and Composite Reliability (CR) indices were examined, ensuring the internal consistency of the measures. Convergent validity was assessed using the Average Variance Extracted (AVE), confirming that the constructs effectively captured the variance of their respective indicators. Discriminant validity was evaluated using the Fornell-Larcker criterion and the Heterotrait-Monotrait ratio (HTMT), ensuring that the constructs were distinct and not overly correlated.

Multicollinearity among the predictors was checked using the Variance Inflation Factor (VIF), ensuring that the predictors were independent and did not distort the results. To test the study’s hypotheses, bootstrapping techniques were applied to evaluate the significance of the path coefficients, providing robust estimates of the relationships in the model. The model’s explanatory power was measured through R², indicating how well the independent variables explained the dependent variable, while Q² assessed the model’s predictive relevance. Additionally, *f²* (effect size) was used to determine the relative impact of each predictor on the dependent variable.

This rigorous data analysis approach ensured the reliability and validity of the study’s findings, providing strong support for the hypotheses and contributing valuable insights into the research model.

## 4. Results

### 4.1. Measurement model assessment

#### 4.1.1. *Construct reliability and validity.*

Establishing the reliability and validity of constructs is critical to the integrity of the measurement model in this study. Reliable and valid constructs ensure that the data accurately reflect the underlying theoretical concepts, which is vital for drawing meaningful conclusions from the research. To achieve this, several assessments were performed, focusing on the outer loadings, internal consistency, convergent validity, and multicollinearity among constructs.

The outer loadings for the indicators of each construct were assessed to establish the measurement model’s reliability and validity. Outer loadings represent the correlation between observed indicators and their corresponding latent constructs. According to Hair et al. [109], outer loadings above 0.70 are considered acceptable, as they indicate that more than 50% of the variance in the indicator is explained by the latent construct. In this study, all outer loadings ranged from 0.707 to 0.866 (see Table 2), surpassing the 0.70 threshold, thereby confirming the indicators’ robust reliability in representing their respective constructs. This strong indicator reliability ensures an accurate measurement of latent variables, minimizing measurement errors and contributing to the validity of the model.

**Table 2:** Reliability and validity metrics for key constructs in the study.

Constructs			Items	Loadings	CA	CR	AVE	VIF
**SMMAs**	INT	INT1	“Information sharing is possible on the social media sites of brand X.”	0.780	0.765	0.865	0.682	1.388
		INT2	“Expression of opinions is easy on the social media sites of brand X.”	0.849				1.720
		INT3	“The social media sites of brand X interact regularly with its followers and fans.”	0.846				1.726
	CUS	CUS1	“The information that I need can be found in the social media of brand X.”	0.800	0.755	0.860	0.672	1.482
		CUS2	“I feel that my needs are satisﬁed using the social media sites of brand X.”	0.856				1.734
		CUS3	“The social media sites of brand X facilitate a personalized search for information.”	0.803				1.470
	TRE	TRE1	“The information shared in the social media sites of brand X is up to date.”	0.707	0.758	0.861	0.675	1.375
		TRE2	“Content visible on the social media sites of brand X is the latest trend.”	0.731				1.652
		TRE3	“Anything trendy is available on the social media sites of brand X.”	0.740				1.706
	ENT	ENT1	“The social media sites of brand X are enjoyable.”	0.845	0.786	0.875	0.700	1.717
		ENT2	“Utilizing the social media sites of brand X is exciting.”	0.832				1.663
		ENT3	“The content shared on the social media sites of brand X seems interesting.”	0.832				1.566
**PQ**	PQ1		“Organic cosmetics have a benefit that suits my needs.”	0.866	0.807	0.886	0.722	1.821
	PQ2		“Organic cosmetics are better in quality than general cosmetics.”	0.828				1.699
	PQ3		“I am happy with the quality of organic cosmetics.”	0.854				1.752
**PV**	PV1		“Organic cosmetics performance meets my expectations.”	0.821	0.755	0.860	0.671	1.506
	PV2		“Organic cosmetics represent excellent value for money.”	0.816				1.502
	PV3		“Overall, organic cosmetics deliver me good value.”	0.821				1.547
**EWO**	EWO1		“I will recommend these organic cosmetics to others through social media platforms.”	0.856	0.792	0.878	0.706	1.722
	EWO2		“I will be proud to tell others that I use these organic cosmetics through social media platforms.”	0.863				1.731
	EWO3		“I will speak favorably of these organic cosmetics to others through social media platforms.”	0.801				1.590
**PI**	PI1		“I will buy organic cosmetics in the future.”	0.813	0.830	0.887	0.662	1.734
	PI2		“I desire to buy organic cosmetics in the future.”	0.837				1.879
	PI3		“I am likely to buy organic cosmetics in the future.”	0.811				1.838
	PI4		“I plan to buy organic cosmetics in the future.”	0.793				1.707

To ensure the internal consistency of the constructs, Cronbach’s Alpha (CA) and composite reliability (CR) were assessed. Both metrics are widely used to evaluate the reliability of measurement models by determining how closely related the items are within a construct. Cronbach’s Alpha provides a lower-bound estimate of the internal consistency, while composite reliability offers a more accurate measure in the context of structural equation modeling [109]. In this study, as presented in Table 2, Cronbach’s Alpha values ranged from 0.755 to 0.902, and composite reliability values ranged from 0.860 to 0.918, exceeding the 0.70 benchmark recommended by Nunnally and Bernstein [110]. These findings indicate strong internal consistency across all constructs, implying that the items reliably measure the same underlying concepts.

To further assess the model’s validity, convergent validity, which assesses the degree to which items measuring the same construct are correlated, was evaluated using Average Variance Extracted (AVE). According to Fornell and Larcker [111], an AVE value above 0.5 indicates that a construct explains more than half of the variance of its indicators, confirming convergent validity. In this study, as shown in Table 2, AVE values for all constructs—interaction (0.682), customization (0.672), trendiness (0.675), entertainment (0.700), perceived quality (0.722), perceived value (0.671), electronic word-of-mouth (0.706), and purchase intention (0.662)—exceeded the threshold of 0.5, confirming strong convergent validity. This means that the items within each construct are highly correlated, accurately capturing the underlying latent variables.

Moreover, Variance Inflation Factor (VIF) analysis was conducted to assess collinearity within the regression model. VIF values greater than 5 suggest problematic multicollinearity among independent variables, which can inflate standard errors and distort the significance of regression coefficients [112]. In this study, all VIF values were below the recommended threshold, ranging from 1.375 to 1.879, indicating that multicollinearity was not a concern (see Table 2). The low VIF values support the reliability of the path coefficients, ensuring that the relationships between constructs in the structural model are not compromised by excessive correlations among predictors.

*Notes* SMMAs = Social Media Marketing Activities; INT = Interaction; CUS = Customization; TRE = Trendiness; ENT = Entertainment; PQ = Perceived Quality; PV = Perceived Value; EWO = Electronic Word-of-Mouth; PI = Purchase Intention.

Overall, the constructs in the measurement model demonstrate excellent reliability and validity. The high outer loadings, strong internal consistency, and robust convergent validity, combined with the absence of multicollinearity, confirm that the latent variables are well-measured and accurately represent consumer behaviors and attitudes in the context of social media marketing for organic cosmetics.

#### 4.1.2. *Discriminant validity.*

Discriminant validity is an important feature of construct validity that ensures that each construct in the model captures a unique concept and is not excessively correlated with other constructs [113]. This validation step is essential for ensuring that the constructs provide meaningful and accurate insights in the context of the research. To assess discriminant validity, the study employed the Fornell-Larcker criterion, a widely used method in structural equation modeling [111]. According to this criterion, the square root of the average variance extracted (AVE) for each construct should be larger than its correlation with any other construct in the model. In this study, as detailed in Table 3, the square root of the AVE for each construct exceeded its correlations with any other construct, confirming that each construct was distinct and not overlapping with others. This finding indicates that each construct is capturing a unique aspect of the respondents’ perceptions or behaviors and is not overlapping with other latent variables.

**Table 3:** Fornell-larcker criterion for discriminant validity assessment.

Construct	EWO	PI	PQ	PV	SMMAs
**EWO**	0.840				
**PI**	0.592	0.814			
**PQ**	0.613	0.654	0.850		
**PV**	0.629	0.697	0.677	0.819	
**SMMAs**	0.758	0.698	0.726	0.729	0.842
*Notes*. EWO = Electronic Word-of-Mouth; PI = Purchase Intention; PQ = Perceived Quality; PV = Perceived Value; SMMAs = Social Media Marketing Activities					

To further validate the discriminant validity, the Heterotrait-Monotrait (HTMT) ratio was examined. The HTMT ratio is considered a more demanding test of discriminant validity compared to the Fornell-Larcker criterion, as it assesses the correlations between constructs and ensures that they are not excessively related [114]. The general rule of thumb is that HTMT values should be below 0.90 for concepts that are expected to be distinct. In this study, all HTMT ratios were between 0.723 and 0.898 (see Table 4), which were below the threshold of 0.90, confirming that all constructs were adequately differentiated from each other. The low HTMT ratios reinforce the idea that the constructs are not variations of one another but instead represent unique theoretical concepts.

**Table 4:** Heterotrait-Monotrait (HTMT) ratio for discriminant validity assessment.

Construct	EWO	PI	PQ	PV	SMMAs
**EWO**					
**PI**	0.723				
**PQ**	0.761	0.791			
**PV**	0.808	0.876	0.864		
**SMMAs**	0.898	0.806	0.849	0.883	

*Notes* EWO = Electronic Word-of-Mouth; PI = Purchase Intention*;* PQ = Perceived Quality; PV = Perceived Value; SMMAs = Social Media Marketing Activities.

Thus, the discriminant validity of the model was robustly supported by both the Fornell-Larcker criterion and the HTMT ratio. The constructs in the model, including interaction, customization, trendiness, entertainment, electronic word-of-mouth, perceived quality, perceived value, and purchase intention, were confirmed to measure distinct dimensions of the consumer experience. This comprehensive validation ensures that the model’s structure is sound and that the constructs provide meaningful insights into how consumers interact with organic cosmetics in a social media marketing context.

### 4.2. *Structural model assessment*

#### 4.2.1. *Hypothesis testing.*

**Fig 2:**
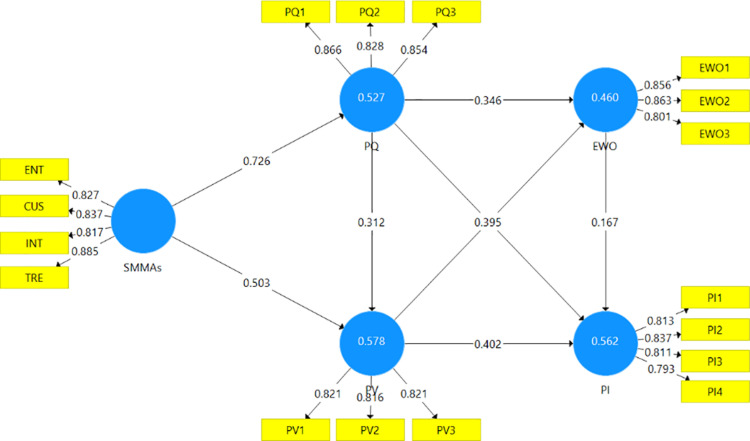
Results of PLS-SEM Analysis.

The structural model assessment is crucial for understanding how the latent constructs interact and influence one another within the research framework [112]. This assessment examines the path coefficients and p-value, which represent the strength and significance of the associations between latent variables. In this study, the path coefficients and p-value reveal key insights into how social media marketing activities (SMMAs) affect perceived quality, perceived value, electronic word-of-mouth (eWOM), and purchase intention in the context of Generation Z’s engagement with organic cosmetics. The results of hypothesis testing are presented in Table 5 and illustrated in Fig 2.

**Table 5:** Hypothesis testing results.

Hypothesis	Paths	Path Coefficient (β)	Stander Deviation (S. D)	t-statistics	p-values	Results
**H1**	SMMAs - > PQ	0.726	0.036	20.117	0.000	Accepted
**H2**	SMMAs - > PV	0.503	0.056	8.914	0.000	Accepted
**H3**	PQ - > PV	0.312	0.060	5.211	0.000	Accepted
**H4**	PQ - > EWO	0.346	0.082	4.200	0.000	Accepted
**H5**	PQ - > PI	0.279	0.072	3.904	0.000	Accepted
**H6**	PV - > EWO	0.395	0.081	4.899	0.000	Accepted
**H7**	PV - > PI	0.402	0.065	6.172	0.000	Accepted
**H8**	EWO - > PI	0.167	0.059	2.819	0.005	Accepted

*Notes*. SMMAs = Social Media Marketing Activities; PQ = Perceived Quality; PV = Perceived Value; PI = Purchase Intention; EWO = Electronic Word-of-Mouth.

The results confirm the first hypothesis, with a significant path coefficient of β = 0.726, p < 0.001, showing that SMMAs significantly influence perceived quality. This suggests that the way social media marketing engages users through interaction, customization, trendiness, and entertainment strongly contributes to how Generation Z perceives the quality of organic cosmetics. This finding highlights that brands that invest in well-executed social media content can effectively enhance perceptions of product quality, which is critical in the cosmetics market, where trust and quality assurance are key factors for consumers. Similarly, the second hypothesis, which posited that SMMAs significantly positively influence perceived value, is supported with a path coefficient of β = 0.503, p < 0.001. This finding indicates that social media marketing significantly contributes to how Generation Z perceives the value of organic cosmetics. This relationship suggests that well-crafted social media campaigns, particularly those that offer customization and relevance, enhance consumers’ perceptions of value. In turn, social media becomes even more important as a tool for increasing product appeal and differentiating organic cosmetics in a competitive market.

Regarding the influences of perceived quality, the results show that perceived quality significantly influences perceived value, with a path coefficient of β = 0.312, p < 0.001, supporting the third hypothesis. This suggests that Generation Z consumers’ perception of a product’s value rises when they believe the organic cosmetics are of high quality. This interconnectedness between quality and value is particularly relevant in the cosmetics industry, where product quality serves as a critical determinant of perceived value. The findings underscore the importance of improving quality perceptions through strategic social media marketing efforts to elevate perceived value among consumers. Additionally, the study supports the fourth hypothesis, with a positive path coefficient of β = 0.346, p < 0.001, indicating that perceived quality significantly influences electronic word-of-mouth (eWOM). This means that when Generation Z consumers perceive the quality of organic cosmetics to be great, they are more willing to share their experiences and recommendations online. This highlights the vital role of product quality in fostering positive eWOM, a powerful driver of consumer behavior in digital environments. High-quality products are thus crucial in generating organic promotion through social media platforms, which can significantly enhance brand visibility and consumer trust. Moreover, the result validates the fifth hypothesis, with the coefficient of β = 0.279, p < 0.001, demonstrating that higher perceived quality directly enhances the intent to purchase. This implies that Generation Z customers are more likely to purchase organic cosmetics when they perceive the products as high-quality. This finding emphasizes the critical importance of maintaining and communicating superior product quality to influence the purchasing decisions of quality-conscious consumers. Thus, perceived quality not only fosters positive eWOM but also directly motivates purchase behavior, making it a cornerstone of successful marketing strategies in the organic cosmetics sector.

Furthermore, the sixth hypothesis is also accepted, indicating that perceived value positively influences eWOM, with a path coefficient of β = 0.395, p < 0.001. The result suggests that consumers who perceive higher value in organic cosmetics are more likely to share their experiences online, further reinforcing the brand’s reputation. This finding emphasizes the importance of perceived value in generating consumer-driven content, which is a key driver of product visibility and appeal on social media platforms. Perceived value also significantly impacts purchase intention, confirming the seventh hypothesis (β = 0.402, p < 0.001). This indicates that Generation Z’s purchasing decisions are heavily influenced by their assessment of value, which could stem from product benefits, ethical considerations, or price-quality trade-offs. Brands that successfully communicate value are likely to see higher purchase intentions from value-conscious consumers.

Finally, the confirmation of the eighth hypothesis reveals that eWOM has a significant positive impact on purchase intention, with a path coefficient of β = 0.167, p < 0.05. This finding underscores the influential role of positive eWOM in driving purchase decisions among Generation Z consumers. Given their strong reliance on peer reviews and social media recommendations, this result highlights how crucial it is for brands to generate favorable eWOM through strategic social media marketing efforts. By fostering positive online conversations and recommendations, brands can effectively enhance purchase intentions within this highly connected and digitally savvy demographic.

#### 4.2.2. *Explanatory and predictive power.*

The explanatory and predictive power of the research model was evaluated using R-squared (R²) and Q-squared (Q²) values, respectively. R² values measure the model’s ability to explain the variance in dependent variables, while Q² values, derived from the blindfolding procedure, assess its predictive accuracy [115–117].

The R² values indicate the model’s explanatory power by showing the percentage of variance in each dependent variable explained by the independent variables [115]. In this study, as shown in Table 6, the R² for perceived quality (PQ) is 0.526, meaning that 52.6% of its variance is explained by social media marketing activities (SMMAs). This indicates that SMMAs, particularly customization and entertainment, have a moderate effect on Generation Z’s perception of organic cosmetics’ quality, showing that these marketing elements play a key role in creating perceptions of product quality. For perceived value (PV), the R² is 0.576, indicating that 57.6% of its variance is explained by perceived quality and SMMAs, with customization being a particularly strong driver. This underscores the importance of both quality perceptions and personalized marketing in shaping how Generation Z evaluates the value of organic cosmetics. The R² for eWOM is 0.457, meaning that 45.7% of the variance in eWOM is explained by perceived quality and perceived value. This suggests that higher perceptions of quality and value significantly increase the likelihood of positive eWOM, as consumers tend to share favorable opinions when they perceive the product to be of high quality and value. Finally, purchase intention (PI) has a high R² of 0.558, indicating that 55.8% of its variance is explained by perceived value and eWOM. This suggests that these two factors substantially influence Generation Z’s intention to purchase organic cosmetics, emphasizing their critical role in the decision-making process. According to Cohen [115], R² values above 0.26 represent a large effect size, confirming the significant impact of the constructs on perceived quality, perceived value, and purchase intention.

**Table 6:** R² and Q² Results.

	R^2^	Q²
**EWO**	0.457	0.315
**PI**	0.558	0.356
**PQ**	0.526	0.369
**PV**	0.576	0.376

*Notes* EWO = Electronic Word-of-Mouth; PI = Purchase Intention*;* PQ = Perceived Quality; PV = Perceived Value.

Q² values assess the model’s ability to predict outcomes accurately. A Q² value greater than zero indicates that the model has predictive relevance for a given construct [116,117]. In this study, as presented in Table 6, the Q² value for perceived quality (PQ) is 0.369, demonstrating that the model has strong predictive relevance for how Generation Z perceives the quality of organic cosmetics based on SMMAs. For perceived value (PV), the Q² value is 0.376, reflecting the model’s high predictive power in forecasting perceived value, based on inputs from SMMAs and perceived quality. This indicates that the model accurately predicts how marketing activities and quality perceptions shape value perceptions. The Q² value for eWOM is 0.315, showing that the model can reliably predict eWOM behavior, driven by consumer perceptions of quality and value. This suggests that the model can forecast how positive word-of-mouth is influenced by these perceptions. Finally, purchase intention (PI) has a Q² value of 0.356, indicating strong predictive relevance in forecasting Generation Z’s intent to purchase organic cosmetics, driven by perceived value and eWOM.

The combination of strong R² and positive Q² values suggests that the model not only fits the data well but also holds substantial predictive power for key consumer behaviors. The significant explanatory power shows that SMMAs, perceived quality, and perceived value are critical drivers of Generation Z’s purchase intentions. Meanwhile, the positive Q² values reinforce the model’s predictive relevance, particularly for essential outcomes like purchase intention. These results demonstrate that the model is both theoretically sound and practically valuable for understanding and forecasting consumer behaviors in the context of organic cosmetics.

#### 4.2.3. *Effect size.*

The F-square (ƒ²) effect size is a key metric in structural equation modeling that evaluates the practical significance of predictor variables on dependent variables. It measures the strength of the relationship between constructs beyond statistical significance, helping to assess whether an independent variable meaningfully contributes to explaining the variance in a dependent variable. According to Cohen [115], effect sizes are categorized as small (ƒ² = 0.02), medium (ƒ² = 0.15), and large (ƒ² = 0.35). A higher F-square value indicates a stronger effect, while a lower value suggests a minimal influence of the predictor variable.

The F-square results of this study, presented in Table 7, reveal the impact of various factors on consumer behavior in the organic cosmetics market. Social Media Marketing Activities (SMMAs) exhibit the strongest effect on perceived quality (PQ) (ƒ² = 1.114) and a notable influence on perceived value (PV) (ƒ² = 0.283), highlighting the pivotal role of digital marketing in shaping consumer perceptions. Perceived quality (PQ) has a moderate influence on purchase intention (PI) (ƒ² = 0.135) and a smaller effect on perceived value (ƒ² = 0.109), indicating that while product quality contributes to purchasing decisions, its role in shaping perceived value is comparatively weaker. Additionally, perceived quality (PQ) also has a minor effect on electronic word-of-mouth (EWO) (ƒ² = 0.071), implying that superior product quality can slightly enhance word-of-mouth recommendations. Perceived value (PV) significantly impacts purchase intention (PI) (ƒ² = 0.261) and has a small effect on electronic word-of-mouth (EWO) (ƒ² = 0.074), suggesting that a higher perceived value not only drives purchase decisions but also encourages consumers to share their experiences online. Electronic word-of-mouth (EWO) has the smallest impact on purchase intention (PI) (ƒ² = 0.035), reflecting its relatively weaker direct influence on purchase decisions. These findings emphasize the dominant role of SMMAs in shaping consumer perceptions, which subsequently drive purchase intention and consumer advocacy through electronic word-of-mouth.

**Table 7:** F-square results.

**H1**	SMMAs → PQ	1.114
**H2**	SMMAs → PV	0.283
**H3**	PQ → PV	0.109
**H4**	PQ → EWO	0.071
**H5**	PQ → PI	0.135
**H6**	PV → EWO	0.074
**H7**	PV → PI	0.261
**H8**	EWO → PI	0.035

*Notes*. SMMAs = Social Media Marketing Activities; PQ = Perceived Quality; PV = Perceived Value; PI = Purchase Intention; EWO = Electronic Word-of-Mouth

## 5. Discussion

This study sheds light on the intricate link between Social Media Marketing Activities (SMMAs) and Generation Z’s purchase intentions and electronic word-of-mouth (eWOM) for organic cosmetics in Vietnam, with perceived quality and value acting as mediating factors. The acceptance of all eight hypotheses underscores the intricate dynamics at play and provides significant implications for both researchers and practitioners in digital marketing, consumer behavior, and the emerging organic cosmetics industry.

The study results support hypotheses H1 and H2, confirming that SMMAs significantly enhance both perceived quality and perceived value. This aligns with recent research by Cheung et al. [45], who demonstrated that social media engagement can elevate perceived quality across various product categories. Additionally, Le et al. [118] observed that social media content significantly influenced quality perceptions among Vietnamese consumers in the beauty industry. Together, these findings indicate that well-executed social media campaigns can effectively communicate the premium nature and advantages of organic cosmetics to Generation Z consumers in Vietnam. The positive influence of SMMAs on perceived value (H2) corroborates the work of Rahman et al. [51] and Li et al. [119], who highlighted the role of social media in conveying the long-term benefits and sustainability aspects of eco-friendly products. For organic cosmetics, this implies that SMMAs can effectively underscore both personal and broader environmental benefits, resonating with the values of Generation Z consumers [53]. However, the greater impact of SMMAs on perceived quality compared to perceived value suggests that digital marketing efforts are more effective at shaping consumer trust in product excellence rather than altering value perceptions. One possible explanation is that perceived quality is based on tangible, verifiable product attributes (e.g., ingredients, efficacy, certifications), which social media can effectively communicate through expert reviews, influencer endorsements, and product demonstrations [120]. In contrast, perceived value is a more subjective construct that also depends on pricing and personal financial considerations, which are harder to control through marketing alone [121]. These insights underscore the necessity of designing social media content to emphasize quality and value propositions that align with the preferences and concerns of Generation Z consumers.

Moreover, the acceptance of H3, which posits that perceived quality positively influences perceived value, is consistent with studies focused on emerging markets. For instance, Osburg et al. [59] found that consumers in such markets, including Vietnam, prioritize quality attributes when evaluating product value. This is particularly relevant for organic cosmetics, where perceptions of quality are closely linked to ingredients, efficacy, and safety. Supporting this notion, Hudayah et al. [60] and Zhuang et al. [73] demonstrated that perceived quality is a significant predictor of perceived value among Generation Z consumers, especially concerning green products. Therefore, maintaining high-quality standards is vital for enhancing perceived value in the eyes of young consumers. Given the prevalence of counterfeit beauty products in Vietnam, ensuring credibility through third-party certifications and transparent ingredient sourcing is essential in reinforcing this relationship.

Furthermore, the acceptance of hypotheses H4, H5, H6, and H7 underscores the pivotal roles of perceived quality and value in shaping both eWOM and purchase intention, aligning with contemporary research on organic and green products. Regarding eWOM, the positive influence of perceived quality (H4) is supported by findings from Vergura et al. [122] and Xiao et al. [67], which assert that high-quality product information on social media significantly enhances eWOM among Generation Z. Similarly, the positive impact of perceived value on eWOM (H6) aligns with research by Ahn and Lee [123] and Nguyen et al. [77], emphasizing that when consumers perceive high value in a product, they are more likely to share positive experiences online. This suggests that organic cosmetics brands should integrate user-generated content (UGC) campaigns, where satisfied customers share testimonials and product reviews, thereby amplifying eWOM and enhancing brand credibility. The direct positive influences of perceived quality (H5) and perceived value (H7) on purchase intention are consistent with recent literature. Research by Limbu and Ahamed [23] and Zhuang et al. [73] highlights that both quality and value perceptions are critical determinants of purchase intention within the green cosmetics sector. In Vietnam, where Generation Z consumers are highly engaged with social media but also price-sensitive [11], brands may benefit from combining high-quality messaging with educational content that justifies premium pricing—such as comparisons between organic and non-organic products or long-term benefits for skin health. These findings suggest that marketers should prioritize communicating both quality attributes and value propositions of organic cosmetics to effectively enhance purchase intentions among Generation Z consumers in Vietnam.

Finally, the study confirms that eWOM has a significant positive influence on purchase intention (H8), aligning with a growing body of research that emphasizes the role of online reviews and recommendations in consumer decision-making processes. Studies by Ismagilova et al. [85] and Nofal et al. [124] have demonstrated that the structure, credibility, and quality of eWOM significantly impact Generation Z’s purchase intentions. In the context of organic cosmetics in Vietnam, this finding suggests that fostering positive eWOM as a key strategy to drive customer purchase intentions. Given the dominance of platforms like TikTok, Instagram, and Facebook in Vietnam, brands should optimize their digital presence by encouraging interactive discussions, leveraging short-form video testimonials, and fostering online communities where consumers can exchange product experiences in real time.

When compared to similar studies conducted in other geographies or industries, these findings reveal both commonalities and unique insights. For example, while studies in Western markets have also shown SMMAs’ impact on purchase intention through mediators like brand image or trust [125], this study reveals how cultural factors specific to Vietnam may shape perceptions differently. Similarly, research in industries such as electronics has shown SMMAs’ influence on consumer behavior but often emphasizes different mediators like usability or financial trustworthiness rather than sustainability or eco-consciousness [126]. These comparisons contextualize this study’s findings within a broader framework while underscoring their relevance to industries prioritizing eco-friendly products.

Additionally, this study refines existing theoretical frameworks by extending their application to Generation Z’s behavior toward organic cosmetics. For example, while prior research has often focused on general consumer groups or traditional product categories [127], this study demonstrates how SMMAs, specifically interaction, customization, trendiness, and entertainment, which interact with values like sustainability to influence younger consumers. This refinement enhances our understanding of how value-attitude-behavior (VAB) hierarchy can be adapted to account for generational differences in digital marketing contexts. This research showcases that Gen Z’s core values directly influence their attitudes toward organic cosmetics, which subsequently shapes their purchasing behaviors. This understanding of the alignment of values, attitudes, and behavior for organic cosmetics, contributes to a more nuanced view of the generational impact on these consumer choices.

## 6. Implications

### 6.1. Theoretical implications

This study makes a substantial contribution to the existing literature by presenting a comprehensive model that integrates Social Media Marketing Activities (SMMAs), perceived quality, perceived value, electronic Word-of-Mouth (eWOM), and purchase intention within the context of organic cosmetics for Generation Z consumers in Vietnam. The findings expand understanding of how SMMAs influence consumer perceptions and behaviors in emerging markets, particularly for eco-friendly products. This comprehensive method sheds light on the intricate relationships among these factors, offering essential insights into the complex decision-making processes of young consumers in the rapidly evolving Vietnamese organic cosmetics market. The study fills a crucial gap in the existing literature by investigating the combined effects of these variables in the specific context of organic cosmetics in Vietnam. This focus allows for a more precise understanding of consumer behavior in emerging markets, particularly in the beauty and personal care sector. Concentrating for Generation Z in Vietnam, but certain limitations exist. The focus on Generation Z, the study sheds light on the unique characteristics and preferences of this influential demographic, which is increasingly shaping market trends and driving demand for sustainable products. Furthermore, the study’s findings contribute to the growing body of research on sustainable consumption and green marketing. Through exploring how SMMAs influence perceptions of eco-friendly products, the research provides valuable insights into how digital marketing strategies can effectively promote sustainable consumption behaviors. This is especially pertinent in emerging markets, where growing environmental awareness is leading consumers to become more mindful of the ecological consequences of their buying choices.

### 6.2. Practical implications

In addition to its theoretical implications, this study also provides tremendously useful practical implications to numerous stakeholders. For marketers and businesses in Vietnam’s organic cosmetics industry, this study offers valuable insights for strategic decision-making. The significant influence of Social Media Marketing Activities (SMMAs) on perceived quality and value highlights the need for effective social media strategies. These should emphasize product quality and value propositions, leveraging social platforms to build brand credibility and consumer trust. The strong link between perceived quality and value underscores the importance of maintaining high-quality standards and effectively communicating these to consumers. This communication should highlight the superior quality of organic cosmetics and educate consumers about their long-term benefits for personal health and environmental sustainability. The positive impact of perceived quality and value on electronic word-of-mouth (eWOM) and purchase intention suggests that marketers should focus on enhancing these perceptions. This could involve implementing transparent quality control processes, obtaining relevant certifications, and consistently meeting or exceeding consumer expectations. Brands should also consider developing value-added services or loyalty programs to enhance their overall value proposition. The significant role of eWOM in driving purchase intentions emphasizes the importance of managing customer reviews and recommendations on social media. Brands should actively engage with customers, encourage them to share experiences, and respond promptly to feedback. A robust social media listening strategy can help companies address concerns quickly and capitalize on positive sentiment. Lastly, the focus on Generation Z consumers in Vietnam provides insights for targeting this demographic. Marketers should align their messaging and offerings with this generation’s values, emphasizing sustainability, authenticity, and social responsibility. This may involve collaborating with influencers, creating interactive content, and leveraging popular social media platforms among younger consumers.

## 7. Limitations and recommendations

This research offers important insights into the organic cosmetics market for Generation Z in Vietnam, but certain limitations exist. The focus on this specific demographic and region limits the applicability of the findings to other age groups or regions. Future research could expand to other generations, such as Millennials or Generation X, and explore different emerging markets to capture cultural and economic influences on consumer behavior in the organic cosmetics sector. Additionally, this study’s broad focus on organic cosmetics overlooks potential differences among sub-categories like skincare, makeup, or hair care. Future studies could explore these specific product types to uncover more targeted marketing strategies. The cross-sectional nature of this research also restricts the ability to track changes over time. Longitudinal studies would offer deeper insights into evolving consumer attitudes and the long-term impact of social media marketing and eWOM. Another limitation is the exclusive focus on social media marketing. Exploring the interaction between social media and other channels, such as in-store experiences or traditional media, could provide a more comprehensive view of marketing effectiveness. Finally, the quantitative approach, while robust, may not fully capture the complexity of consumer motivations. Future research could integrate qualitative methods, such as interviews or focus groups, to delve into underlying consumer behaviors. Additionally, investigating the effects of negative eWOM could reveal strategies for managing brand perceptions in the organic cosmetics industry.

## 8. Conclusion

This study presents a comprehensive examination of the determinants influencing Generation Z consumers’ purchase intentions and electronic word-of-mouth (eWOM) behavior regarding organic cosmetics in Vietnam. By employing a quantitative research approach, data were collected from 315 participants with an interest in and experience with organic cosmetics through a 5-point Likert scale questionnaire. The survey utilized validated scales to evaluate key constructs, including various dimensions of Social Media Marketing Activities (SMMAs)—such as interaction, customization, trendiness, and entertainment—as well as perceived quality, perceived value, eWOM, and purchase intention. The data analysis was computed using the Partial Least Squares Structural Equation Modeling (PLS-SEM) statistical technique, which revealed several significant insights.

The findings underscore the critical role that SMMAs play in shaping consumer perceptions and behaviors. Specifically, social media marketing efforts were shown to significantly influence both the perceived quality and perceived value of organic cosmetics among Generation Z consumers in Vietnam. This highlights the necessity for brands to develop effective social media strategies that emphasize product quality attributes and value propositions, thereby building brand credibility and fostering consumer trust. Moreover, the research reveals strong relationships between perceived quality, perceived value, eWOM, and purchase intentions. This suggests that maintaining high-quality standards and effectively communicating the value of organic cosmetics are essential for encouraging positive eWOM and facilitating purchase decisions. Brands should prioritize transparent quality control processes, acquire relevant certifications, and consistently deliver products that meet or exceed consumer expectations. The significant impact of eWOM on purchase intentions further emphasizes the need for brands to actively manage and encourage customer reviews and recommendations on social media platforms. Implementing robust social media listening strategies and promptly addressing both positive and negative feedback will enable companies to build brand advocacy and mitigate potential issues.

Beyond the Vietnamese organic cosmetics market, these findings hold broader implications for brands operating in other emerging and global markets. As social media continues to serve as a primary source of product discovery and engagement worldwide, businesses across different regions can leverage SMMAs to enhance consumer trust, shape brand perception, and drive purchase behavior. The study’s insights can be particularly relevant for industries where product quality, sustainability, and ethical sourcing are key drivers of consumer decision-making, such as skincare, wellness products, and sustainable fashion.

From a theoretical perspective, this study advances the understanding of SMMAs by illustrating their role in shaping perceived quality, perceived value, and consumer behavioral outcomes. By integrating social media marketing with consumer perception models, the research extends existing frameworks, reinforcing the idea that external marketing stimuli influence internal cognitive evaluations and subsequent actions. These findings contribute to the broader discourse on digital marketing effectiveness, particularly in the context of younger, socially engaged consumer segments.

While this research contributes valuable insights to the field, it also acknowledges certain limitations that present opportunities for future studies. Expanding the research scope to include diverse age groups, markets, and product subcategories within organic cosmetics could provide a more holistic understanding of consumer behavior in this sector. Furthermore, longitudinal studies and mixed-method approaches could yield deeper insights into the evolving nature of consumer perceptions and behaviors over time. Looking ahead, future research could explore the integration of emerging technologies, such as artificial intelligence (AI)-powered chatbots, augmented reality (AR) shopping experiences, and personalized digital marketing strategies, to further enhance consumer engagement. Additionally, investigating cross-cultural differences in SMMAs’ impact on consumer behavior could provide a more nuanced understanding of how digital marketing strategies can be tailored to different demographic and geographic segments.

In conclusion, this research adds to the expanding field of organic cosmetics marketing, particularly in the context of emerging markets and younger consumers. The findings offer practical implications for marketers and businesses operating in the organic cosmetics industry, emphasizing the importance of leveraging social media, maintaining product quality, effectively communicating value, and fostering positive eWOM. As the organic cosmetics market continues to expand and evolve, comprehending these dynamics will be crucial for brands aiming to establish and maintain a competitive edge in this rapidly changing landscape.

## Supporting information

S1 File Survey questionnaire. (PDF)

S2 File Dataset used in analysis. (XLSX)
