# Supplementary material for: Uncovering the influence of social media marketing activities on Generation Z’s purchase intentions and eWOM for organic cosmetics
Source: PLoS One. 2025 Jun 11;20(6):e0325953. doi: 10.1371/journal.pone.0325953 (PMC12157167; doi:10.1371/journal.pone.0325953)
Supplement: S1 File — (PDF) [file pone.0325953.s001.pdf]

QUESTIONNAIRE

Role of Social Media Marketing Activities (SMMAs) in shaping Generation Z's Purchase Intention and Electronic Word-of-Mouth for Organic Cosmetics

Dear Participant,

We are conducting a study to examine the impact of social media marketing (SMMA) activities on Generation Z purchase intention and electronic word-of-mouth for organic cosmetics in Vietnam with perceived quality and value as mediators. This study aims to understand how social media marketing influences consumer behavior, particularly focusing on Generation Z's perceptions, intentions, and willingness to share their experiences with organic cosmetic products. Your insights will provide valuable data for analyzing the impact of factors such as perceived quality and value in shaping purchase decisions and electronic word-of-mouth communication.

The survey is anonymous, and your responses will be kept strictly confidential, used only for academic purposes. We greatly appreciate your support in contributing to this research.

Regards,

Do you consent to participate in this survey and allow your information to be used for research purposes?

- Yes
- No

I. Screening Section

Organic cosmetics *are personal care products made from natural ingredients that are grown without the use of synthetic fertilizers, pesticides, or genetically modified organisms (GMOs) (Dini, 2024). Example: Aloe vera moisturizers, herbal cleansers, and plant-based makeup, etc.*

Have you ever purchased or used organic cosmetics?

- Yes
- No

II. Demographics Section

2.1 Gender:

- Male
- Female
- Not to specify

2.2 Age:

- 18 to 20 years old
- 21 to 24 years old
- 25 to 29 years old

2.3 What is your monthly income?

- Less than 5 million VND
- From 5 to 10 million VND
- From 10 to 20 million VND
- From 20 to 30 million VND
- More than 30 million

III. Constructs and Items Section

The following questions focus on your perceptions of social media marketing activities and their influence on organic cosmetics. Please indicate your level of agreement with the statements below by selecting a rating from 1 to 5.

1 = Strongly disagree

2 = Disagree

3 = Neutral

4 = Agree

5 = Strongly agree

**Interaction** (Malarvizhi et al., 2022; Yadav & Rahman, 2018)	1	2	3	4	5
1) "Information sharing is possible on the social media sites of brand X."					
2) "Expression of opinions is easy on the social media sites of brand X."					
3) "The social media sites of brand X interact regularly with its followers and fans."					
**Customization** (Yadav & Rahman, 2018)	1	2	3	4	5
1) "The information that I need can be found in the social media of brand X."					
2) "I feel that my needs are satisfied using the social media sites of brand X."					
3) "The social media sites of brand X facilitate a personalized search for information."					

**Trendiness (**Malarvizhi et al., 2022)	1	2	3	4	5
1) "The information shared in the social media sites of brand X is up to date."					
2) "Content visible on the social media sites of brand X is the latest trend."					
3) "Anything trendy is available on the social media sites of brand X."					
**Entertainment** (Bushara et al., 2023; Zarei et al., 2021)	1	2	3	4	5
1) "The social media sites of brand X are enjoyable."					
2) "Utilizing the social media sites of brand X is exciting."					
3) "The content shared on the social media sites of brand X seems interesting."					
**Perceived Quality** (Suhud et al., 2022; Zeithaml, 1988)	1	2	3	4	5
1) "Organic cosmetics have a benefit that suits my needs."					
2) "Organic cosmetics are better in quality than general cosmetics."					
3) "I am happy with the quality of organic cosmetics."					
**Perceived Value** (Bushara et al., 2023); Doszhanov & Ahmad, 2015)	1	2	3	4	5
1) "Organic cosmetics performance meets my expectations."					
2) "Organic cosmetics represent excellent value for money."					
3) "Overall, organic cosmetics deliver me good value."					
**Electronic Word-of-Mouth** (Mim et al., 2022)	1	2	3	4	5
1) "I will recommend these organic cosmetics to others through social media platforms."					
2) "I will be proud to tell others that I use these organic cosmetics through social media platforms."					
3) "I will speak favorably of these organic cosmetics to others through social media platforms."					

**Purchase Intention** (Alalwan, 2018)	1	2	3	4	5
1) “I will buy organic cosmetics in the future.”					
2) “I desire to buy organic cosmetics in the future.”					
3) “I am likely to buy organic cosmetics in the future.”					
4) “I plan to buy organic cosmetics in the future.”					

Thank you very much for your participation!
